# Psychometric evaluation of the Persian version of the diabetic foot self-care questionnaire in Iranian patients with diabetes

**DOI:** 10.1186/s12902-021-00734-5

**Published:** 2021-04-17

**Authors:** Hassan Mahmoodi, Kamel Abdi, Emmanuel Navarro-Flores, Zaniar Karimi, Hamid Sharif Nia, Reza Ghanei Gheshlagh

**Affiliations:** 1 Social Determinants of Health Research Center, Research Institute for Health Development, Kurdistan University of Medical Sciences, Sanandaj, Iran; 2 Nursing Department, Faculty of Medicine, Komar University of Science and Technology, Sulaymaniyah, Kurdistan Region Iraq; 3 Department of Nursing and Podiatry, Faculty of Health Sciences, University of Malaga, Málaga, Spain; 4 Faculty of Nursing, Kurdistan University of Medical Sciences, Sananda, Iran; 5 School of Nursing and Midwifery Amol, Mazandaran University of Medical Sciences, Sari, Iran; 6 Spiritual Health Research Center, Research Institute for Health Development, Kurdistan University of Medical Sciences, Sanandaj, Iran

**Keywords:** Psychometrics, Diabetes, Diabetic foot, Self-care, Diabetic foot self-care

## Abstract

**Background:**

Diabetic foot self-care refers to a group of self-management behaviors that can reduce the incidence of foot ulcers and amputations. It is necessary to have a valid and reliable standard tool to measure foot self-care in diabetic patients. This study aimed to evaluate the psychometric properties of the Persian version of the Diabetic Foot Self-Care Questionnaire of the University of Malaga, Spain (DFSQ-UMA) in Iran.

**Methods:**

This cross-sectional study was conducted with 407 diabetic patients who were selected using a convenient sampling method. Construct validity was assessed by exploratory (with 207 patients) and confirmatory (with 200 patients) factor analyses. Internal consistency was calculated using Cronbach’s alpha and McDonald’s omega coefficients.

**Results:**

In the exploratory factor analysis, three factors with eigenvalues of 3.84, 2.41, and 2.26 were extracted that together explained 56.74% of the total variance of diabetic foot self-care. A Cronbach’s alpha of 0.865 was found for the total instrument.

**Conclusions:**

The Persian version of the DFSQ-UMA has good validity and reliability, and given its good psychometric properties, it can be used in future studies.

## Background

Diabetes is the most common metabolic disease in the world with an increasing prevalence rate. There are currently 366 million people in the world suffering from diabetes, and this number is predicted to reach more than half a billion by 2030 [1]. End-stage renal disease, foot infection, and blindness are common complications of diabetes that severely affect patients’ quality of life [2]. Amputation, hypoglycemic coma, blindness, and progression of DM have been identified as the four most salient consequences of diabetes [3]. Diabetic foot ulcer (DFU) is the most common complication of diabetes in adults which occurs due to neuropathy and decreased peripheral circulation [4]. According to the results of a meta-analysis by Zhang et al., The prevalence of diabetic foot ulcers in the world was 6.3%, which is higher in men than in women and in type II diabetic patients than in type I diabetics [5].

The presence of a diabetic foot ulcer can lead to permanent disability and infection-associated amputation [6]. The cost of treating a diabetic foot ulcer in the United States is $17,500, which can increase to $30,000 if amputation health care is required [7]. According to Jeffcott et al., the annual cost of managing diabetic foot ulcers in the UK is $1.32 billion, which is equivalent to 1% of the total National Health Service budget [8]. The rate of amputation in diabetic patients is 30 to 40 times higher than in non-diabetic patients [9]. Lower extremity amputation as a consequence of diabetes is considered a key indicator of the quality of foot care [10]. The 5-year mortality rate of patients with diabetic foot ulcers is 50%, which is higher than the mortality rate of patients with prostate and breast cancer and Hodgkin’s disease [11, 12]. Because a major part of diabetic care is provided by the patients themselves, successful control of the disease heavily depends on their skills [13]. More than half of diabetic foot ulcers can be prevented with proper education, so providing patients with foot self-management training should be considered a priority in diabetic foot ulcer prevention programs [14–16]. Despite the importance of diabetic foot self-care, the results of various studies have shown that some diabetic patients never examine their feet during the week or never dry their toes after washing their feet [17–19]. All patients at risk of diabetic foot ulcers should examine their feet thoroughly daily, however only half do so [20, 21].

There are many instruments available to measure self-care in patients with diabetes [22–37], but few of them exclusively examine diabetes foot care, and in most of these tools, self-care is examined generally. Instruments that measure diabetes foot care either have a large number of items that reduce the tendency to respond [19] or have not undergone a proper psychometric analysis [38]. A valid and reliable instrument to assess diabetic foot self-care is the DFSQ-UMA developed by Emmanuel Navarro-Flores et al. (2015) that assesses three domains, including footwear and socks, foot care, and self-care (a = 0.89) [6]. Proper numbers of items covering all aspects of diabetic foot self-care have made the Diabetic Foot Self-Care Questionnaire of the University of Malaga (DFSQ-UMA) a commonly-used instrument. This study aims to investigate the psychometric properties of the Persian version of the DFSQ-UMA.

## Methods

### Study and setting

In this cross-sectional study, the psychometric properties of the Persian version of the DFSQ-UMA have been evaluated and reported. This study was performed in the diabetes unit of Sanandaj (Kurdistan province located in western Iran) in 2020.

### Samples

In the present study, 407 individuals participated who were randomly divided into a 207-member group for the EFA and a 200-member group for the CFA. Patients were selected using a convenient sampling method. All patients with type II diabetes whose diagnosis was confirmed and had a record in the diabetes unit were included in the study. Patients with gestational diabetes mellitus, untreated diabetic foot ulcer, cognitive problems, and inability to communicate were excluded from the study.

### Translation process

The translation process was done forward-backward under the supervision of the original designer. First, the instrument was translated from Spanish to Persian by two translators and then translated into Persian by two other translators [39].

### Validity

For face validity, the questionnaire was given to 10 diabetic patients who were selected by convenience sampling to read it and identify vague sentences and phrases. For content validity, 5 experts (an infectious disease specialist, an internist, a nurse, an orthopedist, and a surgeon) were asked to examine the content of the Persian version of the questionnaire [39].

### Data analysis

Exploratory factor analysis with Principal Axis Factoring and Promax rotation was used to evaluate construct validity. Kaiser-Meyer-Olkin (KMO) and Bartlett’s test of sphericity determined the adequacy of sampling [40]. In the CFA, the model fit was examined based on the following fit indices: Goodness of fit index (GFI), Chi-square test (χ^2^), Degrees of freedom (DF), Root mean square error of approximation (RMSEA), Confirmatory fit index (CFI), Standardized root mean square residuals (SRMR), and Tucker-Lewis index (TLI). GFI > 0.90, CFI and TLI > 0.95, RMSEA < 0.06, and SRMR < 0.08 indicate a good fit to the data [39]. Internal consistency was assessed using Cronbach’s alpha and McDonald’s omega coefficients [41, 42]. Exploratory and confirmatory factors analyses were performed with SPSS software version 26 and Amos 17, respectively.

### Ethical considerations

This study was approved by the research ethics committee at Kurdistan University of Medical Sciences (IR.MUK.REC.1398.263). The study procedure was based on the Helsinki Declaration. Before starting the study, the participants were asked whether they were willing to participate, and were given some information about the study objectives. Also, they were reassured that their personal information would remain confidential.

## Results

A total of 407 patients with type 2 diabetes (153 men and 254 women) aged 25–90 years old (M = 53.62, SD = 17.90) participated. The mean duration of disease was 7.4 ± 6.5 years. Further information is provided in Table 1.

**Table 1:** Demographic description of the sample

Variable	N	%	Mean of diabetic foot self-care	*P*
Gender				
Male	153	37.6	59 ± 11	0.283
Female	254	62.4	60.5 ± 10.1	
Educational Level				
Illiterate	96	23.6	55.6 ± 8.5	0.001
Elementary or middle school	78	19.2	59.8 ± 9.7	
High school education	86	21.1	59.9 ± 9	
College education	147	36.1	62.8 ± 11.8	
Employment Status				
Housewife / unemployed	180	44.2	58.7 ± 9	0.009
Retired	37	9.1	58.6 ± 11.5	
Employed	117	28.7	62.9 ± 10.8	
Self-employed	31	7.6	58 ± 12.5	
Other	42	10.4	59.7 ± 11.7	
Marital Status				
Married	349	85.7	59.5 ± 10.1	0.069
Single	58	14.3	62.6 ± 12.2	
Foot ulcer history				
Yes	37	9.1	60.3 ± 12	0.807
No	370	90.9	59.9 ± 10.3	
Medication				
pills	260	63.9	60.3 ± 10.8	0.02
Insulin	111	27.3	58 ± 9.5	
Pills & insulin	36	8.8	63.3 ± 10.2	

The mean score of diabetic foot self-care was higher in literate patients than in illiterate ones, in employed patients than in unemployed ones and housewives, and in patients taking both pills and insulin than in those taking either pills or insulin.

### Construct validity

#### Exploratory factor analysis

Few statements were rewritten during face and content validity examination. According to the EFA results, a KMO of 0.872 was found, and the Bartlett sphericity test was significant (Chi-Square = 2892.098, do = 120, and *p* = 0.0001). Also, three factors, including knowledge of foot hygiene, appropriate use of footwear and socks, and Podiatric self-care were extracted that together explained 56.74% of the total variance (Table 2).

**Table 2:** Exploratory factor analysis of the Persian version of the DFSQ-UMA

Factors	Items^*^	Factor loading	h^2^	% variance	Eigenvalue	Internal consistency
Knowledge of foot hygiene	2	0.988	0.836	25.60	3.84	α = 0.884 Ω = 0.827
	1	0.925	0.747			
	3	0.830	0.694			
	4	0.722	0.596			
	5	0.533	0.486			
	7	0.346	0.289			
Appropriate use of footwear and socks	11	0.755	0.532	16.08	2.41	α = 0.776 Ω = 0.788
	8	0.723	0.464			
	10	0.705	0.504			
	9	0.654	0.453			
Podiatric self-care	16	0.828	0.536	15.06	2.26	α = 0.750 Ω = 0.738
	15	0.815	0.567			
	12	0.483	0.446			
	13	0.437	0.405			
	14	0.306	0.242			

**Fig. 1:**
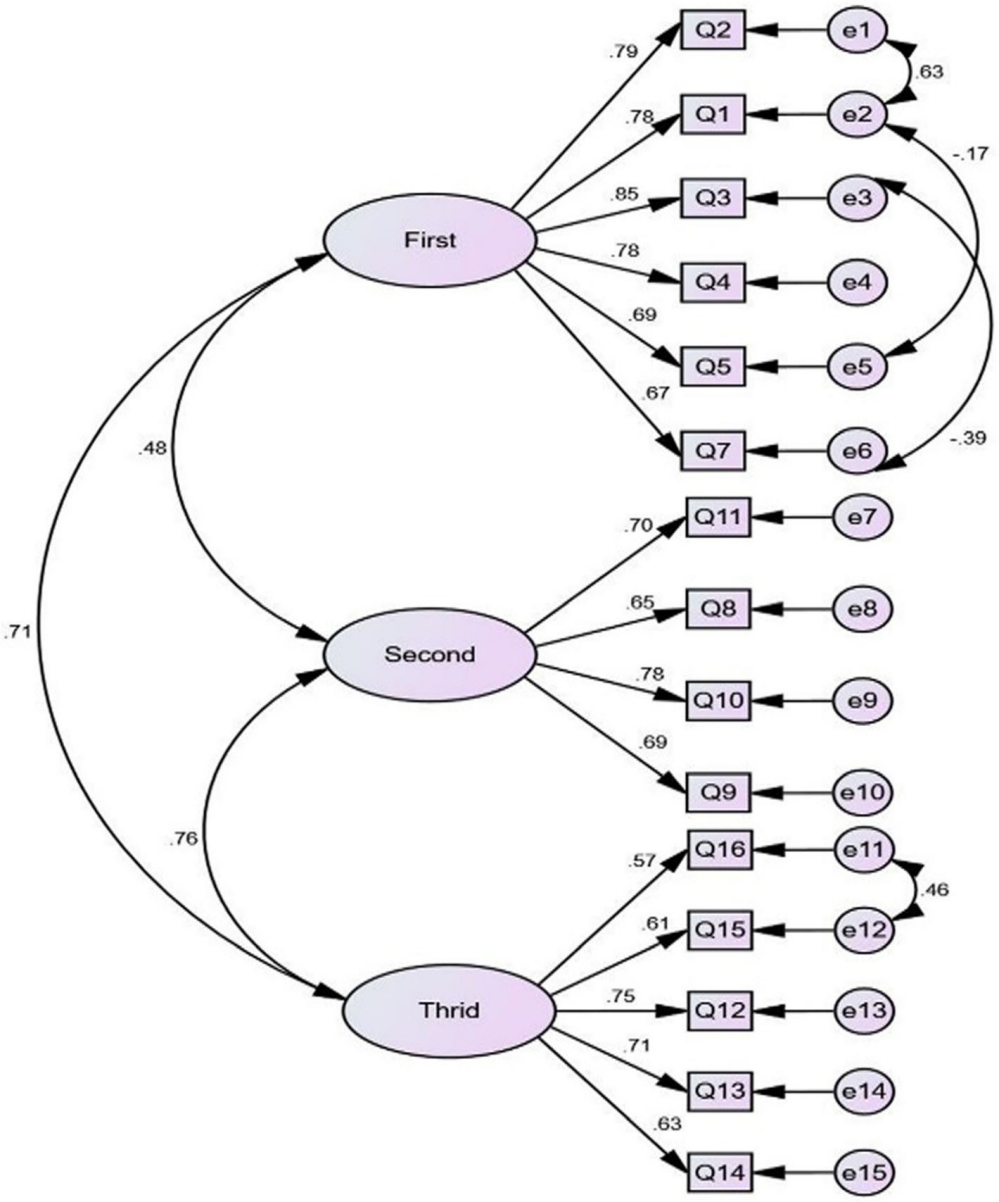
The final model

According to the CFA results (*p* = 0.01, X^2^ = 44.31) the following values were found for the fit indices: Root mean square error of approximation (RMSEA) = 0.049 (90% CI: 0.030–0.067); Comparative fit index (CFI): 0.979; Goodness of fit index (GFI): 0.926; Incremental fit index (IFI): 0.973; Adjusted goodness of fit index (AGFI): 0.893; Parsimony goodness of fit index (PGFI): 0.641; Parsimony comparative of fit index (PCFI): 0.769; Parsimonious normed fit index (PNFI): 0.729; and Minimum discrepancy function by degrees of freedom divided (CMIN/DF) = 1.486 (Fig. 1).

Internal consistency of the questionnaire was examined using Cronbach’s alpha coefficient and McDonald’s omega. According to the results, alphas of 0.884, 0.776, and 0.750 and McDonald’s omega estimates of 0.827, 0.788, and 0.738 were found for knowledge of foot hygiene, footwear, and socks, and Podiatric self-care, respectively that are deemed acceptable.

## Discussion

The purpose of this study was to translate the DFSQ-UMA and examine its psychometric properties. The Persian version of the DFSQ-UMA consists of 15 items. Item #6 of the original version of the questionnaire (What do you use to treat skin lesions, such as dryness and firmness of the skin?) was removed in the CFA. In the EFA, as in the original version, three factors were extracted which together explained 56.74% of the total variance. In the original version, these three factors together explained 60.88% of the total variance [6]. The only difference is that item #7 (how to dry the feet) is in the second factor (Foot-care) of the original version of the questionnaire, while it is in the first factor (Self-care) of the Persian version. Among all items in this factor, item #7 had the lowest factor loading (0.346).

The first dimension consisted of 6 items, which are mostly a kind of self-assessment and focused on a general examination of the feet, examination of wounds, nails, foot hygiene, and drying of the feet. This factor explained the highest percentage of foot self-care and showed the high importance of foot self-care compared to shoes and socks self-care. The results of a study in Tanzania showed that 87% of diabetic patients never examined their feet, and 66% were not interested in learning more about foot care [43]. The second dimension consisted of 4 items that evaluated foot care (shoe selection, nail trimming, foot drying after bathing, and comfortable socks). Checking shoes is important in foot care. In the study by Bell et al. (2005), more than half of the patients did not examine the inside of their shoes before wearing them [17]. The correct technique of nail cutting requires training to prevent the corners of the nails from sinking into the skin of the foot. The results of two different studies in Pakistan [44] and Iran [45] showed that more than half of diabetic patients did not know about this. The third factor had 5 items and referred to the features of conventional shoes, sock texture, new shoe features, summer socks, and warming feet.

In total, this comprehensive tool alone covers all aspects of diabetic foot care (knowledge of foot hygiene, appropriate use of footwear and socks, and podiatric self-care), while previous questionnaires were focused on either foot care or footwear use [19, 46, 47] or knowledge of foot hygiene [38, 48–50].

The mean score of diabetic foot self-care was significantly higher in literate patients (elementary or middle school, high school or high school diploma, and college education) than in illiterate ones. It was also higher in employed patients compared to unemployed ones and housewives. This finding can be attributed to the knowledge and financial independence of this group of patients. The mean score of diabetic foot self-care was also significantly higher in patients taking both pills and insulin than in those taking either pills or insulin. Patients who take both types of drugs simultaneously may consider their condition more serious than others, so they may try to manage it with better self-care. The original version and the Persian version of the questionnaire had Cronbach’s alphas of 0.89 and 0.865, respectively, indicating that both have acceptable reliability [6].

## Conclusions

The DFSQ-UMA is a valid and reliable tool with simple but comprehensive items that can be understood and answered by all patients. The Persian version of this tool can be used to assess foot self-care in Iranian diabetic patients and to plan and implement training programs to promote this ability among patients.

## Data Availability

The datasets used and/or analyzed during the current study are available from the corresponding author on reasonable request.

## Abbreviations

AGFI: Adjusted goodness of fit index
CFA: Confirmatory factor analysis
CFI: Confirmatory fit index
DFSQ-UMA: The Diabetic Foot Self-Care Questionnaire of the University of Malaga
DFU: Diabetic foot ulcer
EFA: Exploratory factor analysis
GFI: Goodness of fit index
IFI: Incremental fit index
KMO: Kaiser-Meyer-Olkin
PCFI: Parsimony comparative of fit Index
PGFI: Parsimony goodness of fit Index
PNFI: Parsimonious normed fit index
RMSEA: Root mean square error of approximation
SRMR: Standardized root mean square residuals
TLI: Tucker-Lewis index

## References

[1] Verrone Quilici MT, Del Fiol FS, Franzin Vieira AE, Toledo MI (2016). Risk factors for foot amputation in patients hospitalized for a diabetic foot infection. J Diabetes Res.

[2] Wukich DK, Raspovic KM, Suder NC (2018). Patients with diabetic foot disease fear major lower-extremity amputation more than death. Foot Ankle Spec.

[3] Quandt SA, Reynolds T, Chapman C, Bell RA, Grzywacz JG, Ip EH, Kirk JK, Arcury TA (2013). Older adults’ fears about diabetes: using common sense models of disease to understand fear origins and implications for self-management. J Appl Gerontol.

[4] Bradbury SE, E Price P. (2011). Diabetic foot ulcer pain: the hidden burden (part two). EWMA J.

[5] Zhang P, Lu J, Jing Y, Tang S, Zhu D, Bi Y (2017). Global epidemiology of diabetic foot ulceration: a systematic review and meta-analysis. Ann Med.

[6] Navarro-Flores E, Morales-Asencio JM, Cervera-Marín JA, Labajos-Manzanares MT, Gijon-Nogueron G (2015). Development, validation and psychometric analysis of the diabetic foot self-care questionnaire of the University of Malaga, Spain (DFSQ-UMA). J Tissue Viability.

[7] Ragnarson Tennvall G, Apelqvist J (2004). Health-economic consequences of diabetic foot lesions. Clin Infect Dis.

[8] Jeffcoate WJ, Vileikyte L, Boyko EJ, Armstrong DG, Boulton AJ (2018). Current challenges and opportunities in the prevention and management of diabetic foot ulcers. Diabetes Care.

[9] Brechow AST, Münch D, Nanning T, Paetzold H, Schwanebeck U, Bornstein S, Weck M (2013). Improving major amputation rates in the multicomplex diabetic foot patient: focus on the severity of peripheral arterial disease. Ther Adv Endocrinol Metab.

[10] van Rensburg JG (2009). Preventative foot care in people with diabetes: quality patient education. J Endocrinol Metab Diabetes S Afr.

[11] Armstrong DGWJ, Robbins JM (2007). Guest editorial: are diabetes-related wounds and amputations worse than cancer?. Int Wound J.

[12] Gazis APN, Macfarlane R, Treece K, Game F, Jeffcoate W (2004). Mortality in patients with diabetic neuropathic osteoarthropathy (Charcot's foot). Diabet Med.

[13] Shahbaz A, Nejad Rahim R (2016). Hemmati Maslak Pak M, Khalkhali HR. the effect of implementing Orem's self-care program on self-care behaviors in patients with a diabetic foot ulcer. J Urmia Nurs Midwifery Fac.

[14] Yazdanpanah L, Nasiri M, Adarvishi S (2015). Literature review on the management of diabetic foot ulcer. World J Diabetes.

[15] Mensing C, Boucher J, Cypress M, Weinger K, Mulcahy K, Barta P (2006). National standards for diabetes self-management education. Diabetes Care.

[16] Gershater MA, Pilhammar E, Apelqvist J, Alm-Roijer C (2011). Patient education for the prevention of diabetic foot ulcers: Interim analysis of a randomized controlled trial due to morbidity and mortality of participants. Eur Diabetes Nurs.

[17] Bell RA, Arcury TA, Snively BM, Smith SL, Stafford JM, Dohanish R, Quandt SA (2005). Diabetes foot self-care practices in a rural, triethnic population. Diabetes Educ.

[18] Pollock R, Unwin N, Connolly V (2004). Knowledge and practice of foot care in people with diabetes. Diabetes Res Clin Pract.

[19] Chin Y-F, Huang T-T (2013). Development and validation of a diabetes foot self-care behavior scale. J Nurs Res.

[20] Wang W, Balamurugan A, Biddle J, Rollins KM (2011). Diabetic neuropathy status and the concerns in underserved rural communities: challenges and opportunities for diabetes educators. Diabetes Educ.

[21] Pinzur MS, Slovenkai MP, Trepman E, Shields NN (2005). Guidelines for diabetic foot care: recommendations endorsed by the diabetes Committee of the American Orthopaedic Foot and Ankle Society. Foot Ankle Int.

[22] Irvine AA, Saunders JT, Blank MB, Carter WR (1990). Validation of scale measuring environmental barriers to diabetes-regimen adherence. Diabetes Care.

[23] Meadows K, Steen N, McColl E, Eccles M, Shiels C, Hewison J, Hutchinson A (1996). The diabetes health profile (DHP): a new instrument for assessing the psychosocial profile of insulin-requiring patients-development and psychometric evaluation. Qual Life Res.

[24] Sturt J, Hearnshaw H, Wakelin M (2010). Validity and reliability of the DMSES UK: a measure of self-efficacy for type 2 diabetes self-management. Prim Health Care Res Dev.

[25] Polonsky WH, Anderson BJ, Lohrer PA, Welch G, Jacobson AM, Aponte JE, Schwartz CE (1995). Assessment of diabetes-related distress. Diabetes Care.

[26] Tu K-S, Barchard K (1993). An assessment of diabetes self-care barriers in older adults. J Community Health Nurs.

[27] Anderson RM, Funnell MM, Fitzgerald JT, Marrero DG (2000). The diabetes empowerment scale: a measure of psychosocial self-efficacy. Diabetes Care.

[28] Fitzgerald JT, Davis WK, Connell CM, Hess GE, Funnell MM, Hiss RG (1996). Development and validation of the diabetes care profile. Eval Health Prof.

[29] Rapley P, Passmore A, Phillips M (2003). Review of the psychometric properties of the diabetes self-efficacy scale: Australian longitudinal study. Nurs Health Sci.

[30] Toobert DJ, Hampson SE, Glasgow RE (2000). The summary of diabetes self-care activities measure: results from 7 studies and a revised scale. Diabetes Care.

[31] Fain JA (2007). Psychometric properties of the Spanish version of the diabetes self-management assessment report tool. Diabetes Educ.

[32] Hearnshaw H, Wright K, Dale J, Sturt J, Vermeire E, Van Royen P (2007). Development and validation of the diabetes obstacles questionnaire (DOQ) to assess obstacles in living with type 2 diabetes. Diabet Med.

[33] Peyrot M, Bushnell DM, Best JH, Martin ML, Cameron A, Patrick DL (2012). Development and validation of the self-management profile for type 2 diabetes (SMP-T2D). Health Qual Life Outcomes.

[34] Stetson B, Schlundt D, Rothschild C, Floyd JE, Rogers W, Mokshagundam SP (2011). Development and validation of the personal diabetes questionnaire (PDQ): a measure of diabetes self-care behaviors, perceptions, and barriers. Diabetes Res Clin Pract.

[35] Welch GW, Jacobson AM, Polonsky WH (1997). The problem areas in diabetes scale: an evaluation of its clinical utility. Diabetes Care.

[36] Tamir O, Wainstein J, Abadi-Korek I, Horowitz E, Shemer J (2012). The patient-perceived difficulty in diabetes treatment (PDDT) scale identifies barriers to care. Diabetes Metab Res Rev.

[37] Brown SA, Becker HA, Garcia AA, Barton SA, Hanis CL (2002). Measuring health beliefs in Spanish-speaking Mexican Americans with type 2 diabetes: adapting an existing instrument. Res Nurs Health.

[38] Lincoln N, Radford K, Game F, Jeffcoate W (2008). Education for secondary prevention of foot ulcers in people with diabetes: a randomized controlled trial. Diabetologia..

[39] Hasanpour Dehkordi A, Chin Y-F, Huang T-T, Ebadi A, Ghanei Gheshlagh R (2020). Psychometric evaluation of the Farsi version of the diabetes foot self-care bahavior scale. J Foot Ankle Res.

[40] Soleimani MA, Yaghoobzadeh A, Bahrami N, Pahlevan Sharif S, Sharif Nia H. Psychometric evaluation of the Persian version of the Templer’s death anxiety scale in cancer patients. Death Stud. 2016;40(9):547–57. 10.1080/07481187.2016.1187688. 10.1080/07481187.2016.1187688 27259574

[41] Dunn TJ, Baguley T, Brunsden V (2014). From alpha to omega: A practical solution to the pervasive problem of internal consistency estimation. Br J Psycho.

[42] Hosseini Z, Eftkhar H, Aghamolaei T, Ebadi A, Nedjat S, Abbasian L (2019). Psychometric properties of the scale for non-adherence to antiretroviral medication (NAME) among HIV-infected patients. Arch Public Health.

[43] Wikblad K, Smide B, Bergström A, Kessi J, Mugusi F (1997). Outcome of clinical foot examination in relation to self-perceived health and glycaemic control in a group of urban Tanzanian diabetic patients. Diabetes Res Clin Pract.

[44] Gondal M, Bano U, Moin S, Masood R, Ahmad A. Evaluation of knowledge and practices of foot care in patients with chronic type 2 diabetes mellitus. J Postgrad Med Inst (Peshawar-Pakistan). 2007;21(2):104–8.

[45] Khamseh ME, Vatankhah N, Baradaran HR (2007). Knowledge and practice of foot care in Iranian people with type 2 diabetes. Int Wound J.

[46] Abetz L, Sutton M, Brady L, McNulty P, Gagnon DD (2002). The diabetic foot ulcer scale (DFS): a quality of life instrument for use in clinical trials. Pract Diabetes Int.

[47] Bann CM, Fehnel SE, Gagnon DD (2003). Development and validation of the diabetic foot ulcer scale-short form (DFS-SF). Pharmacoeconomics..

[48] Carmona GA, Hoffmeyer P, Herrmann F, Vaucher J, Tschopp O, Lacraz A, Vischer UM (2005). Major lower limb amputations in the elderly observed over ten years: the role of diabetes and peripheral arterial disease. Diabetes Metab.

[49] Hinchliffe R, Valk G, Apelqvist J, Armstrong DG, Bakker K, Game F (2008). A systematic review of the effectiveness of interventions to enhance the healing of chronic ulcers of the foot in diabetes. Diabetes Metab Res Rev.

[50] McInnes A, Jeffcoate W, Vileikyte L, Game F, Lucas K, Higson N, Stuart L, Church A, Scanlan J, Anders J (2011). Foot care education in patients with diabetes at low risk of complications: a consensus statement. Diabet Med.

